# MiR-142 inhibits the development of cervical cancer by targeting HMGB1

**DOI:** 10.18632/oncotarget.13136

**Published:** 2016-11-01

**Authors:** Daqiong Jiang, Huiyan Wang, Zhuyan Li, Zhen Li, Xin Chen, Hongbing Cai

**Affiliations:** ^1^ Department of Gynecological Oncology, Zhongnan Hospital of Wuhan University, Hubei Key Laboratory of Tumor Biological Behaviors, Hubei Cancer Clinical Study Center, Wuhan 430071, Hubei, P.R. China; ^2^ Department of Gynecological Oncology, Hospital of Wuhan University of Technology, Wuhan 430070, Hubei, P.R. China

**Keywords:** miR-142, HMGB1, cervical cancer

## Abstract

It has been reported that miRNAs is deregulated in diverse human cancers, involving human cervical cancer. However, the clinical significances and potential mechanisms of miR-142 in the development and progression of cervical cancer were not elucidated completely till now. In this study, we found that the expression of miR- 142 was obviously down-regulated in human cervical cancer tissues and a panel of cell lines. According to statistics, the expression of miR-142 was negatively related to advanced FIGO stage and lymphatic metastasis (*p* < 0.001). Furthermore, our functional analysis revealed the overexpression of miR-142 affected cell proliferation and invasiveness, and enhanced cell apoptosis in representative SiHa and HeLa cells. Based on the molecular level, our findings showed the 3′ untranslated region (3′-UTR) of high-mobility group box 1 protein (HMGB1) was a direct target of miR-142, and determined an inverse correlation with the expression of miR-142. Ectopic expression of HMGB1 could attenuate the inhibitory impact of miR-142 on the proliferation and invasiveness of cervical cancer cells. In conclusion, the present work suggested that miR-142 affects cervical cancer cell proliferation and invasiveness, and enhances cell apoptosis via directly targeting the expression of HMGB1, and these findings may lay a novel foundation for the promising therapy target of cervical cancer.

## INTRODUCTION

Cervical cancer is reported as a leading cause in tumor-related death in the worldwide. Statistically, cervical cancer accounts for most of women cancers [1, 2]. In spite of current progressions in the diagnosis and treatment including operation intervention, chemo-/radiotherapy, the five years overall survival status remains about fifty percent because the diagnosis of most of cervical cancer patients are defined at a advanced stage, and patients had lymphatic metastasis of cervical cancer [3, 4]. Increasing evidence have demonstrated that many signaling pathways affected the development of cervical cancer, among the imbalance between oncogenes and tumor suppressor genes is responsible for the development of tumors [5], however the specific molecular mechanism of cervical cancer is not yet fully elucidated. Ergo, it is crucial to seek prognostic biomarkers and therapy targets of cervical cancer patietns.

MicroRNAs have been thought as a kind of about 17~22 nucleotide noncoding RNAs, which controlled the expression of related genes by binding the 3′-untranslated region (3′-UTR) of target genes based on the post-transcriptional level [6, 7]. Emerging studies have suggested that miRNAs are extensively deregulated in varieties of tumors, and exert anti-tumor or oncogenic role in biological progression of tumors, involving cell proliferation, apoptosis, motility, and invasiveness [8–10]. In recent years, some studies have demonstrated that miR-142 could be recommended as a tumor suppressor in gastric carcinoma, breast carcinoma, and lung cancer [11– 15], indicating that miR-142 might act as a powerful and useful therapy target for the treatment of cancer patients. The previous reports revealed that low expression of miR-142 was found in some cancer tissues, and the expression of miR-142 was significantly related to pathological indicators of cancer patients, and was involved in tumor biology by inhibition of related genes. For example, overexpression of miR-142 affected prostate cancer cell growth via targeting its androgen receptor. However, little is reported about the biological mechanisms of miR-142 in cervical cancer.

In the present study, we found that the expression of miR-142 was significantly down-regulated in cervical cancer tissues and cells, and miR-142 mimics up-regulated the expression of miR-142, and then miR-142 promoted cancer cell proliferation and invasion. What is more, the 3′-UTR of HMGB1 was verified as a target gene of miR-142, and miR-142 inhibited the expression of HMGB1. These findings suggested that miR-142 acts as a tumor-suppressing gene in the development of cervical cancer by directly targeting HMGB1, which will lay a new foundation for the target therapy of cervical cancer patients.

## RESULTS

### MiR-142 is obviously altered in cervical cancer tissues and cells

**Figure 1:**
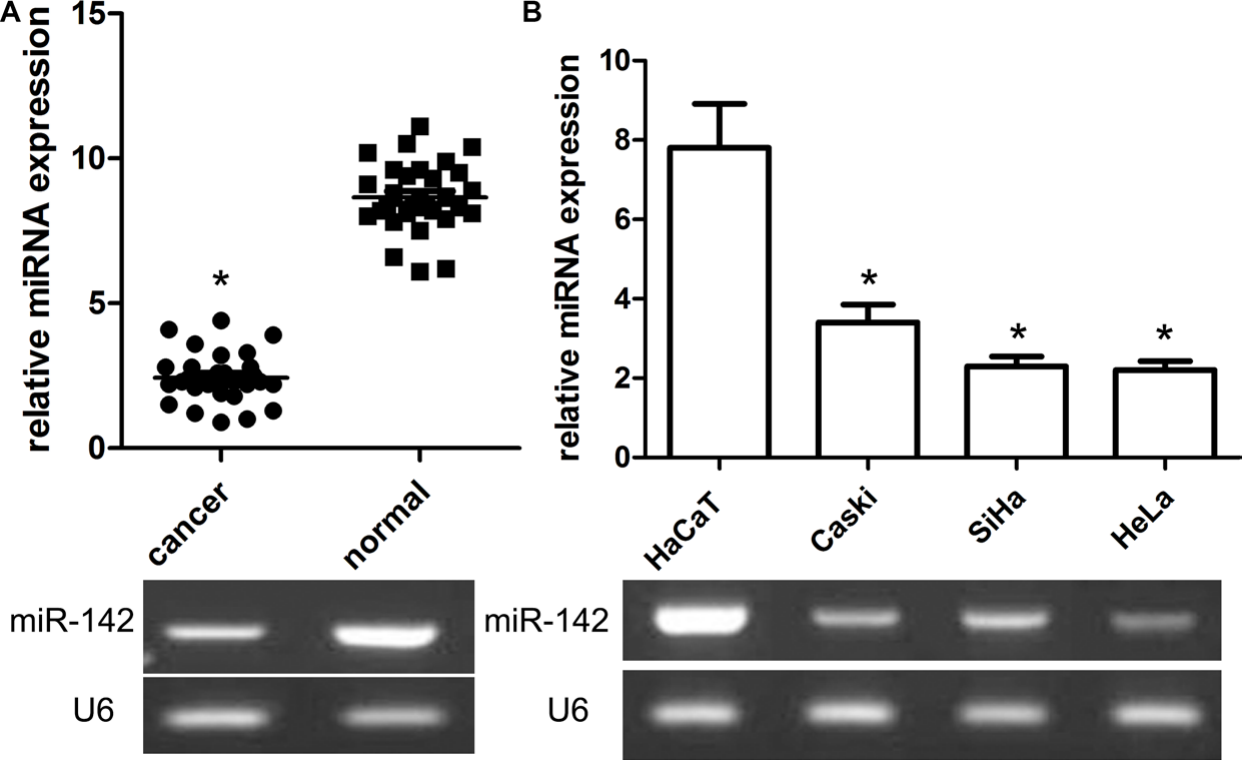
Relative expression of miR-142 in cervical cancer tissues and cell lines as well as its correlation with overall survival of cervical cancer patients (**A**) MiR-142 expression was measured by qPCR and normalized to U6 expression in 30 paired cervical cancer tissues, *P < 0.001, Student's *T-test*. (**B**) qPCR of miR-142 expression in SiHa, and HeLa cells and and normalized to U6 expression, *P < 0.001, Student's *T-test*.

To elucidate the important role of miR-142 in the development of cervical cancer, the expression of miR- 142 was obviously highly expressed in 30 cases of cervical cancer and adjacent non-tumor cervical samples in view of the results of qRT-PCR. In the present work, the findings of our team identified that the expression of miR- 142 was extremely decreased in cervical cancer samples when compared with adjacent normalr cervical samples (*P* < 0.001, Figure 1A). Consistent with these findings, our team further measured the expression status of miR-142 in cervical cancer cells Caski, SiHa, HeLa, and human non-tumor keratinocyte line HaCaT by way of qRT-PCR. The results revealed that a panel of cervical cancer cell lines all exhibited the lower expression level of miR-142 compared with human non-tumor keratinocyte line HaCaT (*P* < 0.01, Figure 1B). Additionally, patients of cervical cancer were divided into low and high expression groups. Based on analysis of clinical pathology characteristics, our results identified that the low miR- 142 expression was related to FIGO stage (*P* = 0.012) and lymphatic metastasis (*P* = 0.023). Unfortunately, we did not find a significant relationship between miR-142 expression and other clinical characteristics including age, grade, and BMI (all *P* > 0.05).

### Ectopic miR-142 expression affects cell proliferation and invasion in cervical cancer cells

**Figure 2:**
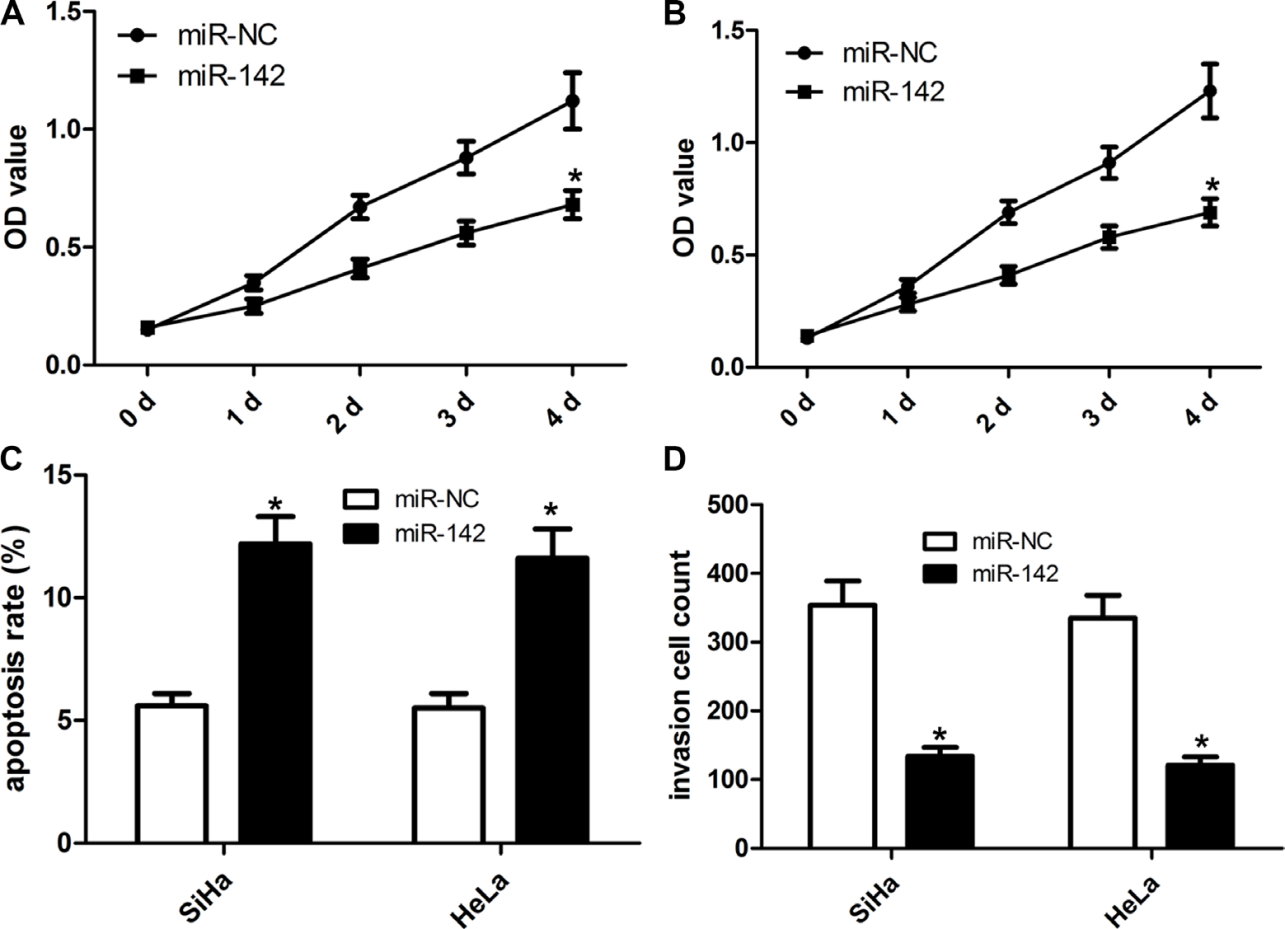
Ectopic miR-142 expression inhibits cell proliferation, invasion and induces cell apoptosis of cervical cancer cells Cell proliferation was detected in SiHa (**A**) and HeLa (**B**) cells that were transfected with miR-142 mimics or mi-NC. (**C**) Cell apoptosis was detected by flow cytometry. Cell apoptotic rate was counted according to summation of the second quadrant and fourth quadrant. (**D**) Matrigel invasion assays were carried out to assess invasive property of cervical cancer cell. The number of invaded cells was calculated under the microscope, mean ± SD, *P < 0.001, Student's *T-test*.

To elucidate the impact of miR-142 on the progression and development of cervical cancer, our team made a miR-142 overexpression vector. The cervical cancer cell lines SiHa and HeLa were identified to over-express miR-142 or miR-NC (negative control). As illustrated in Figure 2, compared with miR-NC, the over-expression of miR-142 significantly affected the growth capacity of SiHa and HeLa cells in a time-dependent fashion. Using cell apoptosis analysis, we found that ectopic miR-142 expression induced the apoptosis of SiHa and HeLa cells (Figure 2A–2B). Transwell cell invasion assay revealed that ectopic expression of miR-142 markedly repressed the invasion capacity of SiHa and HeLa cells (Figure 2C–2D).

### HMGB1 is a direct target of miR-142 in cervical cancer

**Figure 3:**
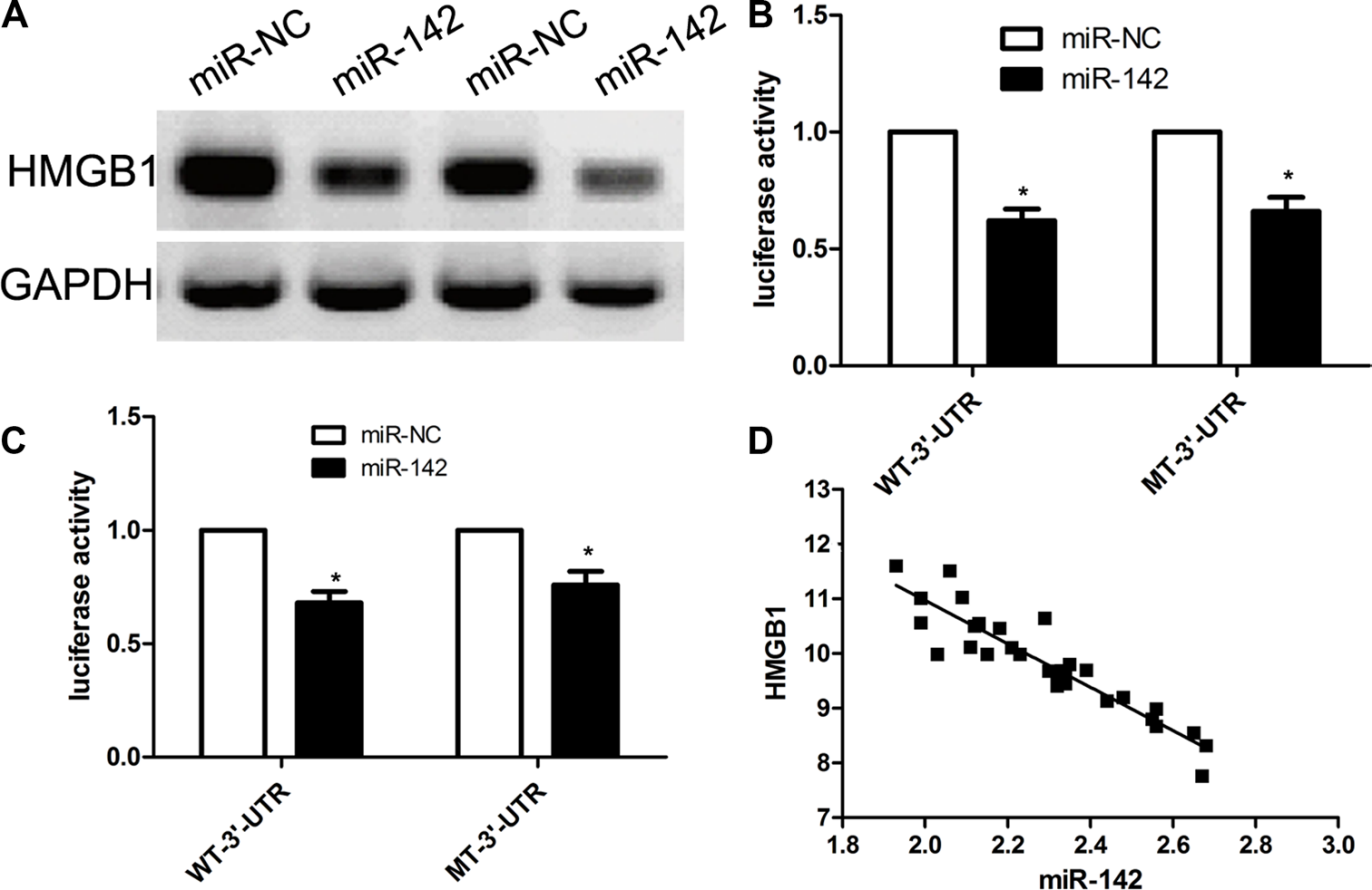
HMGB1 is a direct target of MiR-142 in cervical cancer cell lines (**A**) miR-142 binding sites is located in the 3′-UTR of HMGB1 mRNA. (**B**) The wild type and mutant type of 3′-UTR reporter vectors and control group were co-transfected into cervical cancer SiHa cells with miR-142 or miR-NC. The relative luciferase activities were detected. (**C**) Western blot measure HMGB1 expression in cervical cancer cells. (**D**) According to Pearson's correlation analysis, the expression of HMGB1 was inversely associated with the expression of miR-142. All assays were performed in triplicate, mean ± SD, *P < 0.001, Student's *T-test*.

To further out the potential molecular mechanisms underlying miR-142-induced inhibition of cervical cancer biology, we used the bioinformatic tools to predict potential target genes of miR-142 using the free TargetScan. According to bioinformatics calculation, our team found that HMGB1 can act as a direct target of miR-142 (Figure 3A). In view of these results, our team made a hypothesis that HMGB1 gene might be implicated in biological processes of miR-142 in the progressions of cervical cancer. To validate our hypothesis, our team firstly conducted a dual luciferase assay and identified that miR-142 overexpression obviously inhibited the luciferase activity of wt-3′UTR of HMGB1, however miR- 142 overexpression did not affect the luciferase activity of mut-3′UTR of HMGB1 in both cervical cancer cells (Figure 3B). Subsequently, our team investigated the impact of miR-142 on transcription and expression of HMGB1 gene. Our findings revealed that the expression level of HMGB1 protein was obviously decreased in miR- 142 over-expression group when compared with miR-NC group in SiHa and HeLa cells (Figure 3C). To further validate this result, our team investigated the expression levels of HMGB1 in 70 cases of cervical cancer samples using qPCR. We found that miR-142 expression was negatively correlated with the expression of HMGB1 based on the results from 30 cases of tumor tissues (R2 = − 0.851, *p* < 0.001) (Figure 3D). All in all, our data suggests that HMGB1 might become a potential target of miR-142.

### HMGB1 is implicated in miR-142-induced cell proliferation, invasiveness and apoptosis of cervical cancer cells

**Figure 4:**
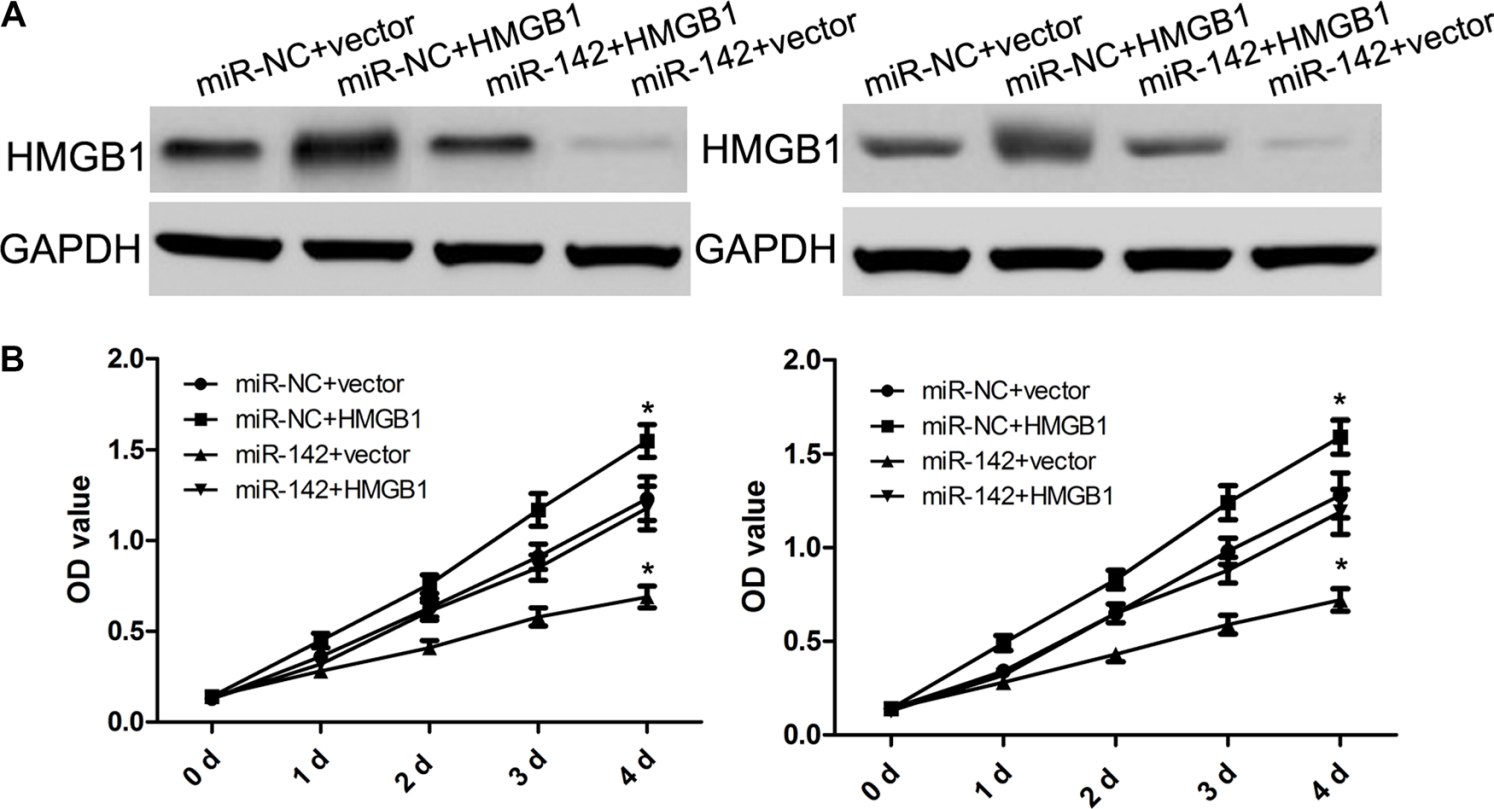
HMGB1 over-expression attenuates the suppressive effect of miR-142 on cell proliferation (**A**) Western blot analysis of HMGB1 protein expression in cervical cancer cells with miR-NC or miR-142 transfected with either pcDNA3.1 or pcDNA3.1-HMGB1, GAPDH was used as a loading control. (**B**) CCK-8 assay detecting the proliferation of cervical cancer cells with different treatments.

**Figure 5:**
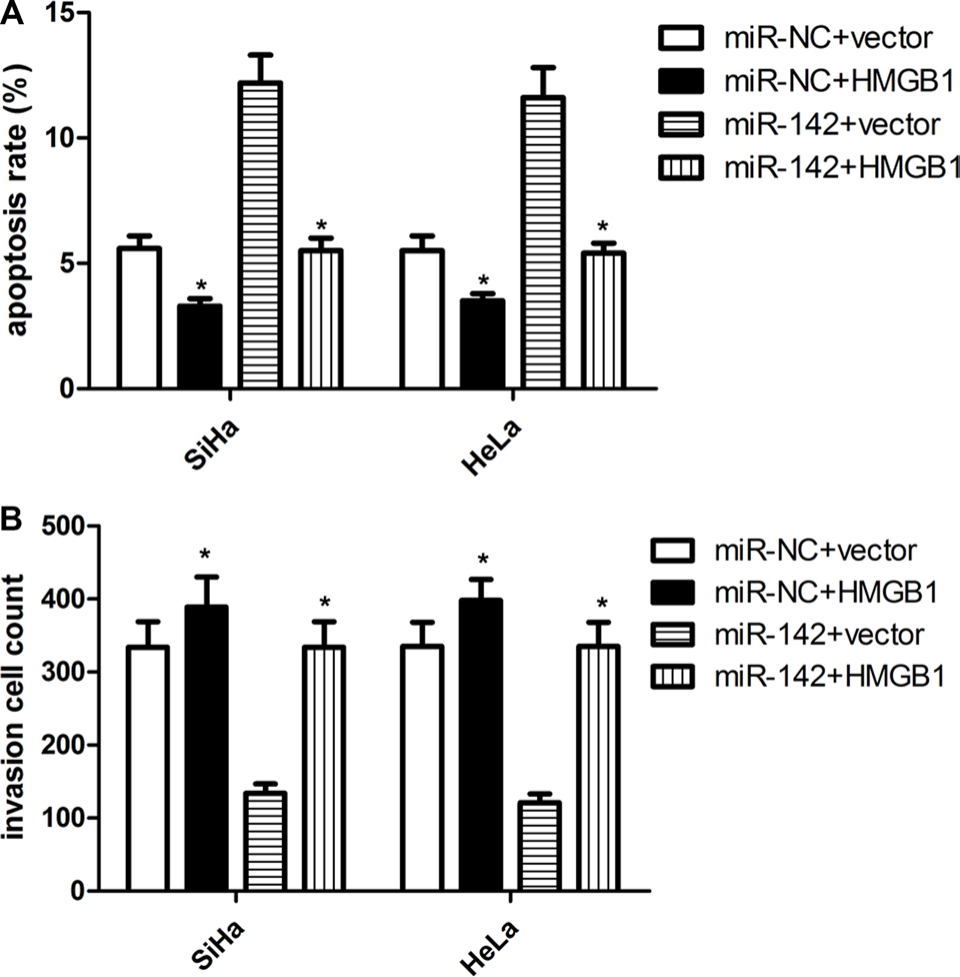
HMGB1 over-expression attenuates the suppressive effect of miR-142 on cell apoptosis and invasion (**A**) Cell apoptosis was detected in cervical cancer cells with different treatments. (**B**) Matrigel invasion assay evaluating invasion of cervical cancer cells with different treatments. Each assay was repeated three times. *P < 0.001, Student's *T-test*.

To characterize the biological role of miR-142 in targeting HMGB1 gene, miR-142 mimics or miR-NC were individually co-transfected into SiHa and HeLa cells with HMGB1 over-expression or negative control. Western blot analysis identified that ectopic HMGB1 expression reverse its inhibition by miR-142 in some degree (Figure 4A). Furthermore, analyses results from proliferation and invasion assays indicated that the overexpression of HMGB1 dramatically restored cell proliferation and invasion inhibited by miR-142 in SiHa and HeLa cells (Figures 4 and 5). In addition, over- expressed HMGB1 also had a similar opposite effect on SiHa and HeLa cell apoptosis mediated by miR-142 (Figure 5). These findings indeed suggested that the role of miR-142 in cervical cancer cells is based on its regulation of HMGB1 expression.

## DISCUSSION

In recent years, it has been reported that miR-142 and HMGB1 is involved in the progression of tumors by binding to their target genes [16–20]. Besides, cancer metastasis also accounts for a main factor in cancer-related deaths [21]. Therefore, interference with tumor progression or metastasis will be a useful method for the treatment of cancers. It is critical to screen useful markers for interference with cancer metastasis, prognosis and individualized therapy of cervical cancer patients. In this study, we provide important evidence in support of miR- 142 functioning as a tumor suppressor in CC. We found that miR-142 suppressed proliferation and invasion of cancer cells both *in vitro*. We further identified that miR-142 targeted at HMGB1 and suppressed their expression at translation level in CC cells. In addition, we found aberrant expression of HMGB1 were significantly associated with lymphatic metastasis of CC patients. Taken together with previous results, this study presents supplementary evidence that overexpression of miR-142 could suppress proliferation and invasion of CC cells by targeting 3′-UTRs of HMGB1. Thus, miR-142 should become a promising novel target due to its diagnostic and prognostic value for cervical cancer.

Emerging evidence demonstrated miRNA as a biomarker, and miRNA-targeting precise treatment will become a hot topic for malignancies. The altered expression of miRNAs has been reported in the development of cervical cancer, and related to poor prognosis. For instance, some well-known miRNAs, including miR-22, miR-260, miR-37b, are widely reported as a diagnostic or prognostic factor for cervical cancer patients [22–24]. Up to now, miR-142 has been studied in some cancers, and exerts an obvious anti-tumor effect on tumor proliferation, just consistent with blocking of related genes [18]. In this work, our team identified that low expression of miR-142 can be found in a majority of cervical cancer tissues, and the potential mechanisms may be attributed to deregulation of generation of miR-142. Importantly, our correlation analysis identified that low miR-142 expression had no significant association with clinical features, like age, grade. All things considered, our data indicates that altered miR-142 expression is a common event in patients of cervical cancer, and altered miR-142 expression may be implicated into malignancy of cervical cancer.

Next, our team demonstrated HMGB1 gene as a direct target of miR-142 in cervical cancer cells, and verified that the inhibitory effect of miR-142 on cervical cancer cells was mediated by the regulation of expression of HMGB1. HMGB1 has been demonstrated to be over-expressed in cervical cancer tissues [15–17]. Other results identified that HMGB1 can modulate cancer cell biology and epithelial-mesenchymal transition. It should be noted that HMGB1 was also involved in cancer cell growth, invasiveness and apoptosis of cervical cancer. These reports and results indicated that HMGB1 was involved in the development and progression of cervical cancer. In the present study, our team identified that the expression of HMGB1 was decreased due to enhanced expression of miR-142 in cervical cancer cells, and we demonstrated an inverse association between the miR-142 expression and HMGB1 mRNA by detecting cervical cancer tissues. The dual luciferase assay strongly identified that HMGB1 gene was a direct target of miR-142, and HMGB1 could attenuate miR-142-mediated inhibitory effects on the proliferation and invasion of cervical cancer cells as well as the expression of HMGB1 protein. These phenomena indicate that miR-142- HMGB1 pathway may be a potential therapy target in the treatment of cervical cancer.

In conclusion, our present study identified miR-142 as a tumor suppressor in the progression of cervical cancer, including cell growth, invasiveness and apoptosis via its direct target HMGB1. The study demonstrates a potential molecular mechanism underlying the inhibitory effect of miR-142 on cervical cancer, and indicates that miR-142 might act as a potential prognostic biomarker and therapeutic target in cervical cancer patients.

## MATERIALS AND METHODS

### Ethics statement

The use of all tissue specimens was approved by the Hospital Ethics Committee of Zhongnan Hospital of Wuhan University. The entire participant provides their written informed consent to participate in the study.

### Patient samples

Primary tumor tissues and normal tissues were obtained from patients (mean age 57.5 ± 12.5 years, range 19–80 years) enrolled in the Department of Pathology, Zhongnan Hospital of Wuhan University between 2015 and 2016. All samples were obtained with patients’ informed consent and were histologically confirmed by staining with hematoxylin-eosin(HE). The histological grade of cancers was assessed according to criteria set by the American Joint Committee on Cancer (AJCC). None of the patients received radiotherapy or chemotherapy before surgery. The present study was approved by the Hospital Ethical Committee.

### Cell culture

Cervical cancer cell lines (American Type Culture Collection, Manassas, VA) were cultured in Eagle's Minimum Essential Medium (ATCC) supplemented with 5% fetal bovine serum (FBS; Hyclone, Logan, UT) and 0.1% penicillin/streptomycin (Fisher Bioreagents, Pittsburgh, PA). All the cell lines were cultured at 37°C in a 5% CO2 atmosphere incubator with humidity.

### Cell transfection

MiR-142 mimics (sense: 5′-UAGCAGCACAUCAU GGUUUACA-3′, antisense: 5′-UAAACAUGAUGUG CUGCUGUU-3′), control miRNAs (10nmol/l), HMGB1 plasmids and control vector were all obtained from Ambion (Ambion, USA). And then cell transfection was carried out using lipofectamine 2000 (Life Technologies, USA) according to manufacturer's instructions.

### Western blot

The cell lysates were prepared in RIPA buffer (Byotime, Haimen, China), and western blot was performed as previously described. The proteins were then transferred to a nitrocellulose membrane (Bio-Rad, Hercules, CA) and blocked with 5% non-fat milk in TBST for 2 h at room temperature with shaking. The membrane was immunoblotted with a primary antibody (Cell Signaling Technology, Danvers, MA) at 4°C overnight. Signals were developed by using an HRP-linked secondary antibody (1:10000) with the ClarityTM Western ECL Substrate (Bio-Rad, Hercules, CA). The intensity of the signals was determined by the FluorChemTM system (Protein Simple, Santa Clara, CA).

### RNA extraction, retrotranscription and quantitative real-time PCR (qRT-PCR)

Total RNA was isolated from the cells with TRIzol reagent (Invitrogen, Carlsbad, CA,USA) and for human FFPE tissues the total RNA was isolated using the RecoverAllTM Total Nucleic Acid Isolation Kit (Ambion, Austin, TX, USA) according to the manufacturer's protocol. qRT-PCR for 142 and HMGB1 was described in previous reports. qRT-PCR was performed using Takara (Dalian, China) on an FTC-3000TM System (Funglyn Biotech Inc., Toronto, Canada). U6 and GAPDH were used as controls for miRNA and mRNA level, respectively. Relative quantitation was calculated using the 2-ΔΔCt method.

### Dual luciferase activity assay

As for dual luciferase activity assay, the 3′UTR target site could be amplified using PCR system, and then the luciferase reporter constructs were also amplified by PCR, which is the HMGB1 3′UTR and carried a putative miR-142-binding site. Cells were transfected with the reporter constructs and then transfected with miR-142 or control miRNAs using lipofectamine 2000 (Life Technologies, USA). Reporter assays were carried out using the dual-luciferase assay system (Promega) according to the manufacturer's instructions.

### Cell proliferation and invasion assays

CCK-8 assay (Kumamoto, Japan) was used to detect the cell growth status according to manufacturer's instruction. Cell proliferation rate was detected at 0 hour, 24 hour, 48 hour and 72 hour following the treatment. As for cell invasion assay, Cell invasion was determined using 24-well transwell chambers with 8 μm pore size polycarbonate membranes (Corning Incorporated, Corning, NY, USA). 2 × 105 transfected cells were seeded on the top side of the membrane pre-coated with Matrigel (BD, Franklin Lakes, NJ, USA) in DMEM without serum. The lower chambers were filled with DMEM containing 10% FBS as a chemoattractant. After being incubated 24 hours, the non-invasive cells on the top side of the membrane were removed with a cotton swab, and invaded cells on the lower membrane surface were fixed in 20% methanol and then stained with 0.1% crystal violet. Invasion was quantified by counting cells in five randomly selected fields of view in each well under the invert microscope (Olympus, Tokyo, Japan).

### Statistical analysis

All statistical analyses were performed with Graphpad Prism software. The statistical significance between the two groups was determined using Student's *t-test*. All experiments were repeated at least three times. Data were expressed as mean ± SEM. Differences were considered statistically significant at *P* < 0.05.
